# Associations of Vitamin D with Inter- and Intra-Muscular Adipose Tissue and Insulin Resistance in Women with and without Polycystic Ovary Syndrome

**DOI:** 10.3390/nu8120774

**Published:** 2016-11-30

**Authors:** David Scott, Anju Joham, Helena Teede, Melanie Gibson-Helm, Cheryce Harrison, Samantha Cassar, Samantha Hutchison, Peter R. Ebeling, Nigel Stepto, Barbora de Courten

**Affiliations:** 1 Department of Medicine, School of Clinical Sciences at Monash Health, Faculty of Medicine, Nursing and Health Sciences, Monash University, Clayton 3168, Victoria, Australia; peter.ebeling@monash.edu; 2 Melbourne Medical School (Western Campus) and Australian Institute of Musculoskeletal Science, The University of Melbourne and Western Health, St Albans 3021, Victoria, Australia; 3 Monash Centre for Health Research and Implementation, School of Public Health and Preventive Medicine, Monash University, Clayton 3168, Victoria, Australia; anju.joham@monash.edu (A.J.); helena.teede@monash.edu (H.T.); melanie.gibson@monash.edu (M.G.-H.); cheryce.harrison@monash.edu (C.H.); samantha.cassar@vu.edu.au (S.C.); sammyhutch@hotmail.com (S.H.); nigel.stepto@vu.edu.au (N.S.); barbora.decourten@monash.edu (B.d.C.); 4 Diabetes and Vascular Medicine Unit, Monash Health, Clayton 3168, Victoria, Australia; 5 Institute of Sport Exercise and Active Living, Victoria University, Melbourne 8001, Victoria, Australia

**Keywords:** polycystic ovary syndrome, insulin resistance, intramuscular adipose tissue, vitamin D, exercise, obesity

## Abstract

Low vitamin D and insulin resistance are common in polycystic ovary syndrome (PCOS) and associated with higher inter- and intra-muscular adipose tissue (IMAT). We investigated associations between vitamin D, IMAT and insulin resistance in a cross-sectional study of 40 women with PCOS and 30 women without PCOS, and pre- and post-exercise in a 12-week intervention in 16 overweight participants (10 with PCOS and six without PCOS). A non-classical body mass index (BMI) threshold was used to differentiate lean and overweight women (BMI ≥ 27 kg/m^2^). Measurements included plasma 25-hydroxyvitamin D (25OHD), insulin resistance (glucose infusion rate (GIR; mg/m^2^/min), fasting glucose and insulin, and glycated haemoglobin), visceral fat, mid-thigh IMAT (computed tomography) and total body fat (dual-energy X-ray absorptiometry). Women with both PCOS and low 25OHD levels had the lowest GIR (all *p* < 0.05). Higher IMAT was associated with lower 25OHD (B = −3.95; 95% CI −6.86, −1.05) and GIR (B = −21.3; 95% CI −37.16, −5.44) in women with PCOS. Overweight women with pre-exercise 25OHD ≥30 nmol/L had significant increases in GIR, and decreases in total and visceral fat (all *p* < 0.044), but no associations were observed when stratified by PCOS status. Women with PCOS and low 25OHD levels have increased insulin resistance which may be partly explained by higher IMAT. Higher pre-training 25OHD levels may enhance exercise-induced changes in body composition and insulin resistance in overweight women.

## 1. Introduction

Polycystic ovary syndrome (PCOS) is associated with reproductive disorders and cardiometabolic consequences including obesity, type 2 diabetes and cardiovascular disease risk factors [1,2]. Low vitamin D levels are similarly associated with increased obesity and cardiometabolic disorders [3,4]. We have previously reported that vitamin D levels are lower in overweight women with PCOS compared to overweight women without PCOS and that low vitamin D levels are associated with insulin resistance in women with PCOS [5], hypothesising that poor vitamin D levels may contribute to the association between PCOS and poor cardiometabolic health.

We have previously shown that a 12-week moderate to vigorous intensity exercise program failed to normalise insulin sensitivity for overweight women with PCOS compared to overweight women without PCOS [6]. It is possible that low vitamin D levels in women with PCOS contribute to this reduced exercise responsiveness. Low vitamin D levels are associated with reduced fat oxidation during exercise in women [7], and also smaller exercise-associated improvements in body composition and muscle function in older adults [8,9].

Low vitamin D may impact on poor cardiometabolic health and exercise responsiveness via infiltration of skeletal muscle tissue with adipose tissue. Intra- and intermuscular adipose tissue (IMAT) likely contributes to insulin resistance through local secretion of pro-inflammatory adipokines and impaired insulin signalling in muscle [10]. We have previously proposed that low vitamin D status contributes to increased accumulation of IMAT [11], and higher IMAT levels are also associated with poorer exercise responsiveness in older adults [12]. It is therefore possible that the low vitamin D status of women with PCOS contributes to higher IMAT levels, and that IMAT explains blunted improvements in insulin resistance following exercise.

The aims of this study were to determine cross-sectional associations of vitamin D, IMAT and insulin resistance in women with and without PCOS, and to explore whether baseline vitamin D status and IMAT influence changes in insulin resistance in overweight women with PCOS compared to overweight women without PCOS following a 12-week exercise intervention.

## 2. Materials and Methods

### 2.1 Study Design and Participants

This research project consisted of a secondary analysis of a cross-sectional study of overweight and lean women with and without PCOS, in which a subset of overweight women underwent a 12-week exercise intervention [6,13,14]. Participants were recruited through community advertisements. The Monash Health Research Advisory and Ethics Committee approved the study and participants gave written informed consent (RMO 06/6905-2005/091), and the trial was registered (ISRCTN84763265).

Forty-one premenopausal women with PCOS were recruited (18 lean and 23 overweight), as well as 35 women without PCOS (19 lean and 16 overweight). Women were categorised, a priori, as lean or overweight based on a non-classical threshold body mass index (BMI) of 27 kg/m^2^ which aligns with poorer metabolic health [14]. Specifically, this is the inflexion point in the relationship between BMI and insulin resistance [14,15], a key outcome measure for the present study. Diagnosis of PCOS was undertaken by endocrinologists (S.K.H., A.E.J) based on meeting Rotterdam criteria with two of (i) irregular menstrual cycles (21 or 35 days); (ii) clinical (hirsutism, acne) or biochemical (elevation of at least one circulating ovarian androgen) hyperandrogenism and (iii) polycystic ovaries on ultrasound [16]. Hyperprolactinemia, thyroid dysfunction and specific adrenal disorders were excluded clinically and where indicated, biochemically. All women without PCOS had regular menses and no evidence of clinical or biochemical hyperandrogenism. Exclusion criteria were smoking, diabetes, recent weight change of 5 kilograms or more in the previous six months, actively trying to lose weight, and pregnancy.

At screening, standard diet and lifestyle advice were delivered based on recommendations provided by the National Heart Foundation. Participants who were taking medications affecting end-points, including insulin sensitisers, anti- androgens and hormonal contraceptives, ceased these medications with a three-month washout period before baseline. Baseline and post-training data were collected in the follicular phase of the menstrual cycle for controls and whenever feasible in women with PCOS.

### 2.2. Exercise Intervention Subgroup

A subset of overweight and obese (BMI >27 kg/m^2^) inactive (<100 min per week of self-reported moderate and vigorous physical activity) premenopausal women with (*N* = 16) and without (*N* = 13) PCOS underwent an exercise intervention. Participants undertook 12 weeks of supervised, progressive, moderate and vigorous exercise training on a motorised treadmill. Participants attended three 1-h sessions each week, which sequentially alternated between moderate-intensity (20–60 min of walking or jogging at 70% of peak oxygen uptake (VO_2_ peak)) and high-intensity interval training (six to eight five-minute intervals at ~95%–100% of VO_2_ peak with two-minute passive recovery periods). Participants’ exercise was progressively increased during the course of the study. At study completion, baseline testing was repeated.

### 2.3. Anthropometrics and Body Composition

Body weight and height were assessed and BMI calculated as kg/m^2^. Total body fat mass was estimated by a whole-body dual energy X-ray absorptiometry (DXA) scan (GE Lunar Prodigy (GE Lunar Corp., Madison, WI, USA) using operating system version 9). Participants underwent single-slice axial scans at the abdomen (L4–L5 intervertebral disc space level without angulation) and the mid-thigh (mid-point between the anterior iliac crest and the patella) of both legs as previously described [17]. All scans were performed using a GE Lightspeed computed tomography (CT) scanner (GE Medical Systems, Milwaukee, WI, USA) and saved as DICOM images for analysis. Standard CT procedures of 120 kV, 5 mm thickness and a 512 × 512 matrix were used for all participants, and images were analysed using Slice-O-Matic version 4.3 software (Tomovision, Magog, QC, Canada).

In the present study, IMAT was defined as inter-muscular fat (fat between muscles), and muscle density was defined as intramuscular fat (fat within muscles) [10]. Attenuation levels for delineating fat (less than −30 Hounsfield units (HU)) and muscle (−29 to 150 HU) and manual demarcation of muscle from bone and subcutaneous and intermuscular fat were used as previously described [18]. Mean muscle density was determined by averaging all pixels within the range −29 to 150 HU, with higher values indicating lower muscle lipid content.

### 2.4. Cardiometabolic Outcomes

VO_2_ peak was assessed using the MOXUS modular system (AEI Technologies, Pittsburgh, PA, USA) while participants exercised on a treadmill (Biodex RTM 500, New York, NY, USA) until volitional fatigue. The primary outcome for insulin resistance was glucose infusion rate (mg/m^2^/min) assessed by the euglycaemic–hyperinsulinaemic clamp technique as previously described [13]. Clamp timing was standardised to 48 h after exercise, and included a standardised high-carbohydrate diet for 72 h before an overnight fast. Insulin (Actrapid; Novo Nordisk, Bagsvaerd, Denmark) was infused at 40 mU/m^2^·min for 120 min, with plasma glucose maintained at ~5 mmol/L using variable infusion rates of 25% glucose. Glucose infusion rates (GIRs) were calculated during steady state, achieved in the last 30 min of the clamp and expressed as glucose (mg) per body surface area (m^2^) per min.

Plasma 25OHD was determined using, a commercial direct competitive chemiluminescent immunoassay (Liaison, DiaSorin, Stillwater, MN, USA) with a manufacturer-specified analytical range of 10–350 nmol/L and CV 6.5%–10.7%. Stored blood samples (−80 °C) were batch analysed for serum fasting glucose, total cholesterol, high-density lipoprotein (HDL) cholesterol, low-density lipoprotein (LDL) cholesterol, triglycerides, insulin and glycosylated haemoglobin (HbA1c) [14].

### 2.5. Statistical Analyses

Prior to analyses, data were assessed for normality by Shapiro–Wilk tests and log transformed where appropriate. One-way ANOVA with Bonferroni post-hoc comparisons were performed to determine cross-sectional differences in cardiometabolic outcomes according to PCOS and 25OHD levels (≥ or <50 nmol/L) [19]. Multivariable linear regression, stratified by PCOS status, investigated associations of thigh IMAT and muscle density with cardiometabolic outcomes after adjustment for age and visceral fat area.

For the exercise study, paired *t*-tests compared pre- and post-exercise values for total body composition, IMAT and cardiometabolic outcomes in overweight women defined as having lower or higher 25OHD according to the median for this cohort (30 nmol/L). Univariable linear regression analyses, stratified by PCOS status, examined whether pre-training 25OHD, thigh IMAT or muscle density (as continuous variables) were predictors of changes in GIR from pre- to post-training. All statistical analyses were performed using SPSS version 23.0 (IBM, Armonk, NY, USA) and *p*-values < 0.05 were considered statistically significant.

## 3. Results

### 3.1. Cross-Sectional Study of Lean and Overweight Women with and without PCOS

Of 76 women recruited for this study, six had incomplete body composition data and were excluded from the analysis. Amongst 70 overweight and lean participants at baseline, 40 (57%) were diagnosed as having PCOS. There was no difference in prevalence of low 25OHD (<50 nmol/L) for women with and without PCOS (73% vs. 60%, respectively, *p* = 0.271).

There were also no differences in age when participants were classified according to PCOS and 25OHD levels (Table 1). GIR was significantly lower in women with PCOS and low 25OHD compared to all other sub-groups. Women with PCOS and normal 25OHD had significantly lower total fat mass (*p* = 0.005), and higher muscle density (indicating lower fat infiltration of muscle; *p* = 0.025) compared with women with PCOS and low 25OHD. Fasting insulin levels were higher in women with PCOS and low 25OHD compared with women with PCOS and normal 25OHD (*p* = 0.015).

Multivariable linear regression analyses investigated associations of thigh muscle density and IMAT with cardiometabolic parameters in women with and without PCOS (Table 2). Higher IMAT was associated with lower HDL cholesterol in both women with and without PCOS. Lower muscle density was associated with lower HDL in women with PCOS, while lower muscle density was associated with higher LDL cholesterol in women without PCOS. In women with PCOS only, lower muscle density and higher IMAT were both associated with higher triglycerides. Lower muscle density and higher IMAT were also associated with higher fasting insulin in women with PCOS. Lower muscle density was also associated with higher insulin, and additionally with higher HbA1c, in women without PCOS. In women with PCOS, lower muscle density and higher IMAT were both associated with lower GIR and lower 25OHD levels and in women without PCOS, higher IMAT was also associated with lower 25OHD levels (Table 2).

### 3.2. Exercise Intervention in Overweight Women with and without PCOS

Of 29 overweight or obese women who commenced the exercise intervention, sixteen (10 with PCOS, six without PCOS) completed the 12-week exercise intervention and had complete data for pre- and post-training (age 31.9 ± 5.9 years; BMI 36.2 ± 6.0 kg/m^2^ (range 28.3–49.5 kg/m^2^)). Fifteen out of sixteen participants had low 25OHD (<50 nmol/L) pre-training. Table 3 reports pre-training and post-training values for body composition and cardiometabolic parameters stratified by pre-training 25OHD levels of less than, or greater than or equal to, the median 25OHD in this cohort (30 nmol/L). Those with higher 25OHD levels had significant increases in GIR and VO_2_ peak, and significant reductions in total fat and visceral fat, post-training. Comparatively, those with lower 25OHD levels had significant improvements in VO_2_ peak with only a trend towards improved GIR (*p* = 0.07). Similar proportions of women with lower or higher 25OHD levels pre-training had PCOS (57% vs. 67%, respectively, *p* = 0.70).

Univariable linear regression analyses investigated associations of pre-training 25OHD, IMAT and muscle density with indicators of insulin sensitivity stratified by PCOS status. There were no significant associations for women with or without PCOS (all *p* > 0.05).

## 4. Discussion

In our cross-sectional study, women with PCOS and low 25OHD levels had significantly increased insulin resistance compared with counterparts with normal 25OHD, and also those without PCOS. Low 25OHD levels were also associated with higher thigh IMAT, and thigh IMAT was associated with higher triglycerides and insulin, and lower HDL cholesterol and higher insulin resistance. Overweight women with and without PCOS and with higher, but not low, pre-training 25OHD levels demonstrate improvements in body composition and insulin resistance following an exercise intervention.

We have demonstrated that PCOS has an intrinsic insulin resistance irrespective of obesity; and age, BMI, testosterone and sex hormone-binding globulin could not explain all of the insulin resistance [20]. We have also previously demonstrated that 25OHD is associated with insulin resistance in women with PCOS [5], and have expanded our findings here by demonstrating in our cross-sectional study that women with PCOS and low 25OHD status (<50 nmol/L) have significantly increased insulin resistance compared to counterparts without PCOS and/or low 25OHD levels. These findings suggest that vitamin D may play an important role in insulin resistance, particularly in PCOS. In a study of 38 women with PCOS, Muscogiuri and colleagues demonstrated that higher BMI and total body fat mass were the strongest predictors of low 25OHD amongst metabolic and hormonal parameters [21], suggesting that the association of vitamin D with insulin resistance in PCOS may simply reflect increased adiposity. Nevertheless, our previous study indicated that the relationship of low vitamin D with insulin resistance in PCOS women is independent of total body fat mass [5], which might indicate that insulin resistance in women with PCOS and low vitamin D is not attributable to higher fat mass alone.

Total body fat mass may not be a reliable indicator of the extent of ectopic fat depots which are potentially more strongly related to cardiometabolic outcomes than subcutaneous fat depots [22]. Although there were no differences in visceral fat according to PCOS and 25OHD levels in this study, women with PCOS and low 25OHD had significantly lower thigh muscle density (indicating higher fat infiltration) than women with PCOS and normal 25OHD levels. We have previously proposed that vitamin D reduces deposition of IMAT in skeletal muscle because vitamin D deficient states have been shown to induce myoblasts to transdifferentiate into an adipogenic lineage [11]. Our findings are also consistent with previous research reporting that 25OHD levels were significantly associated with lower muscle density in 90 women aged 16 to 22 years [23], with similar findings reported for inter-muscular fat in older adults [24]. Thus, low vitamin D may contribute to increased IMAT, although it is also possible that higher IMAT levels result in decreased circulating vitamin D given that fat tissue acts as a storage site for vitamin D [4]. Furthermore, low vitamin D levels result in increased levels of parathyroid hormone which promotes the influx of calcium into adipocytes [25]. Intracellular calcium in adipocytes may enhance lipogenesis and so this may be another pathway by which low vitamin D status contributes to increases in IMAT and also insulin resistance.

In the cross-sectional cohort, thigh IMAT was positively associated with triglycerides and negatively associated with HDL cholesterol in women with PCOS. Erector spinae IMAT also correlates positively with triglycerides in middle-aged sedentary adults, potentially due to increased inflammation and lipolysis rates in muscle [26]. The lower HDL cholesterol in those with higher thigh IMAT may also reflect that IMAT is lower in more physically active individuals, even amongst those with type 2 diabetes [27]. Erector spinae IMAT has been shown to be associated with higher homeostasis model assessment of insulin resistance (HOMA-IR) and insulin levels in older adults [28], and thigh IMAT is also associated with poorer insulin sensitivity in obese individuals with and without type 2 diabetes [29]. The present study demonstrated a significant association between IMAT and insulin resistance in women with PCOS. Of note, this association was independent of visceral fat area and was not present in women without PCOS. IMAT may potentially have a greater impact on insulin resistance than visceral fat due to its proximity to skeletal muscle, which is the largest insulin sensitive tissue in the body accounting for 85% of glucose utilisation [30]. Indeed, intrinsic abnormalities in glucose transport and insulin-signalling have been observed in cultured skeletal muscle from women with PCOS [31]. It is also possible that the association between IMAT and insulin resistance in women with PCOS, but not those without PCOS, is explained by the higher levels of systemic inflammation associated with PCOS [32], which may contribute to adipose tissue inflammation and dysregulation [33].

In our exercise trial which included overweight women with and without PCOS, we observed that 94% of women had low vitamin D levels (<50 nmol/L). Women with 25OHD levels equal to or above the median (30 nmol/L) for this cohort had significant decreases in fat mass and improvements in insulin sensitivity over 12 weeks that were not observed in women with 25OHD below the median. Our previous research suggests that older adults with higher 25OHD levels combined with higher physical activity have the smallest age-related gains in body fat over five years [8], and this may be related to evidence suggesting that fat oxidation is enhanced during exercise in women with higher vitamin D levels [7]. Further research is needed in this area given the potential clinical implications of enhanced responsiveness to exercise regimens.

Insulin sensitivity can be improved through vitamin D supplementation in individuals with pre-diabetes [34]. Interventions investigating whether vitamin D supplementation can also enhance exercise-induced improvements in metabolic health are necessary in overweight women with PCOS, who demonstrate increased risk for vitamin D deficiency, and who are inherently insulin resistant [2]. A randomised controlled trial of 104 overweight and obese women with PCOS has demonstrated that calcium and vitamin D co-supplementation over eight weeks reduced insulin levels, HOMA-IR and triglycerides and LDL cholesterol levels [35]. However, given that the present study demonstrated no association between pre-training IMAT levels and changes in insulin resistance, or between pre-training vitamin D levels and changes in IMAT, it does not appear that potential benefits of higher vitamin D in PCOS are attributable to lower muscle fat infiltration. Nevertheless, addressing low vitamin D and physical inactivity in women with PCOS may provide significant improvements in quality of life through reductions in adiposity [36]. This may be relevant to other features of PCOS, given that higher vitamin D levels are associated with greater reproductive success in women with PCOS [37], and that there is evidence that vitamin D reduces cytokine secretion through its effects on the NF-κB pathway in adipocytes [38], which may reduce systemic inflammation observed in PCOS.

The findings of this study are subject to limitations. Muscle density is an indirect assessment of muscle lipid content and future studies assessing these relationships would be strengthened by the use of muscle biopsy with assessment of local inflammatory and insulin signaling markers. The strengths of the study include the use of gold-standard assessments for body composition and insulin resistance, and the 90% adherence amongst those who completed the exercise intervention with no significant difference between women with and without PCOS [6].

In conclusion, women with PCOS and low 25OHD levels have increased insulin resistance. This may be partly explained by low 25OHD and the relationship with higher thigh inter- and intra-muscular adipose tissue. We report that overweight women with higher pre-training 25OHD levels demonstrate improvements in body composition and insulin resistance following an exercise intervention. Further research is required to determine potential benefits of vitamin D supplementation for reducing insulin resistance and for potentially enhancing metabolic responses to exercise in women with PCOS.

## Figures and Tables

**Table 1:** Baseline characteristics of participants according to polycstic ovary syndrome (PCOS) and vitamin D status.

Variable	Non-PCOS		PCOS	
	Normal Vitamin D * (*N* = 12)	Low Vitamin D (*N* = 18)	Normal Vitamin D (*N* = 11)	Low Vitamin D (*N* = 29)
Age (y)	29.6 ± 6.8	30.6 ± 6.5	28.5 ± 5.8	28.4 ± 5.0
BMI (kg/m^2^)	26.5 ± 6.4	28.8 ± 8.8	23.4 ± 3.0 ^d^	31.5 ± 8.3 ^c^
Total fat mass (kg)	26.5 ± 14.8	32.0 ± 19.6	19.0 ± 7.5 ^c^	37.8 ± 14.1 ^d^
Visceral fat area (cm^2^)	123.8 ± 23.5	132.2 ± 27.9	143.1 ± 23.5	125.1 ± 54.2
Thigh IMAT area (cm^2^)	3.2 ± 1.6	4.0 ± 2.3	2.8 ± 1.6	4.4 ± 1.6
Thigh muscle density (HU)	50.1 ± 4.8	50.6 ± 3.5	53.5 ± 2.2 ^d^	50.2 ± 2.4 ^c^
25OHD (nmol/L)	69.7 ± 18.9 ^b,d^	32.1 ± 12.4 ^a,c^	63.4 ± 9.1 ^b,d^	32.1 ± 9.5 ^a,c^
Total cholesterol (mmol/L)	4.8 ± 0.7	4.5 ± 0.7	5.2 ± 0.6	4.8 ± 1.0
HDL cholesterol (mmol/L)	1.5 ± 0.4	1.4 ± 0.4 ^c^	1.9 ± 0.4 ^b,d^	1.2 + 0.3 ^c^
LDL cholesterol (mmol/L)	2.9 ± 0.5	2.7 ± 0.7	3.0 ± 0.4	3.1 ± 0.8
Triglycerides (mmol/L)	0.9 ± 0.4	0.9 ± 0.6	0.8 ± 0.3	1.2 ± 0.8
GIR (mg/m^2^/min)	314.6 ± 46.5 ^d^	296.0 ± 97.8 ^d^	296.2 ± 78.2 ^d^	197.2 ± 87.8 ^a,b,c^
Fasting glucose (mmol/L)	4.7 ± 0.3	4.6 ± 0.3	4.5 ± 0.3	4.8 ± 0.5
Log Insulin	1.6 ± 0.4	1.7 ± 0.3	1.5 ± 0.3 ^d^	1.9 ± 0.4 ^c^
HbA1c (%)	5.1 ± 0.2	5.2 ± 0.4	5.0 ± 0.2	5.3 + 0.4
VO_2_ peak (mL/kg/min)	36.4 ± 8.3 ^d^	32.5 ± 10.10 ^a,c^	37.2 ± 7.8 ^d^	28.2 ± 7.2 ^a,c^

BMI: body mass index; IMAT: intermuscular adipose tissue; HU: Hounsfield Units; 25OHD: 25-hydroxyvitamin D; HDL: high-density lipoprotein; LDL: low-density lipoprotein; GIR: glucose infusion rate; HbA1c: glycosylated haemoglobin. ^a^ denotes significantly different (*p* < 0.05) to normal vitamin D, non-PCOS; ^b^ denotes significantly different to low vitamin D, non-PCOS; ^c^ denotes significantly different to normal vitamin D, PCOS; ^d^ denotes significantly different to low vitamin D, PCOS. * Normal vitamin D ≥50 nmol/L; low vitamin D <50 nmol/L.

**Table 2:** Multivariable regression coefficients (95% confidence intervals) for associations of thigh IMAT and muscle density with cardiometabolic health indicators after adjustment for age and visceral fat area.

Variable	No PCOS *N* = 30		PCOS *N* = 40	
	Thigh Muscle Density	Thigh IMAT	Thigh Muscle Density	Thigh IMAT
Total cholesterol (mmol/L)	−0.05 (−0.12, 0.02)	−0.01 (−0.15, 0.13)	0.05 (−0.06, 0.16)	−0.02 (−0.19, 0.15)
HDL cholesterol (mmol/L)	0.01 (−0.04, 0.04)	**−0.08 (−0.15, −0.02)**	**0.07 (0.02, 0.12)**	**−0.11 (−0.19, −0.03)**
LDL cholesterol (mmol/L)	**−0.06 (−0.12, −0.01)**	0.04 (−0.08, 0.16)	0.03 (−0.07, 0.12)	0.01 (−0.14, 0.14)
Triglycerides (mmol/L)	0.03 (−0.03, 0.08)	0.07 (−0.03, 0.17)	**−0.09 (−0.18, −0.01)**	**0.20 (0.08, 0.33)**
GIR (mg/m^2^/min)	5.64 (−2.60, 13.88)	−12.83 (−28.62, 2.95)	10.38 (−0.38, 21.13)	**−21.30 (−37.16, −5.44)**
Fasting glucose (mmol/L)	−0.02 (−0.05, 0.01)	0.03 (−0.02, 0.09)	−0.05 (−0.10, 0.01)	0.06 (−0.03, 0.14)
Log insulin	**−0.04 (−0.06, −0.01)**	0.05 (−0.01, 0.10)	**−0.07 (−0.12, −0.02)**	**0.11 (0.03, 0.18)**
HbA1C (%)	**−0.06 (−0.08, −0.03)**	0.05 (−0.01, 0.11)	−0.02 (−0.06, 0.02)	0.03 (−0.04, 0.09)
25OHD (nmol/L)	−0.65 (−3.13, 1.83)	**−5.67 (−9.94, −1.40)**	**2.94 (1.12, 4.76)**	**−3.95 (−6.86, −1.05)**

Bold values are significant at *p* ≤ 0.05.

**Table 3:** Comparison of pre-training and post-training values for body composition and cardiometabolic parameters according to pre-training vitamin D status.

Variable	25OHD <30 nmol/L * (*N* = 7)			25OHD ≥30 nmol/L (*N* = 9)		
	Pre-Training	Post-Training	*p*-Value	Pre-Training	Post-Training	*p*-Value
Total fat mass (kg)	49.7 ± 12.3	47.4 ± 10.9	0.15	46.1 ± 10.3	44.3 ± 10.7	**0.02**
Visceral fat area (cm^2^)	136.2 ± 51.2	124.9 ± 44.2	0.25	118.1 ± 57.7	111.8 ± 59.4	**0.04**
Thigh IMAT (cm^2^)	4.0 ± 1.2	3.4 ± 1.4	0.16	4.8 ± 1.8	4.7 ± 1.9	0.75
Thigh muscle density (HU)	50.4 ± 1.7	50.3 ± 2.0	0.82	48.6 ± 3.6	48.4 ± 2.9	0.68
GIR (mg/m^2^/min)	183.7 ± 109.4	239.3 ± 137.7	0.07	206.0 ± 99.9	238.9 ± 93.5	**0.04**
Fasting glucose (mmol/L)	5.0 ± 0.5	4.8 ± 0.4	0.13	4.8 ± 0.3	4.8 ± 0.2	0.36
Log Insulin	1.4 ± 0.2	1.4 ± 0.2	0.96	1.3 ± 0.2	1.2 ± 0.3	0.13
HbA1C (%)	5.5 ± 0.4	5.6 ± 0.3	0.41	5.4 ± 0.3	5.5 ± 0.3	0.33
VO_2_ peak (mL/kg/min)	22.7 ± 3.4	27.5 ± 4.8	**< 0.01**	28.5 ± 8.1	37.2 ± 6.1	**< 0.01**
25OHD (nmol/L)	25.6 ± 6.4	26.8 ± 11.2	0.77	38.4 ± 11.9	44.9 ± 15.4	0.22

* Median 25OHD level in this cohort of overweight women with (*N* = 10) and without (*N* = 6) PCOS. Bold values are significant at *p* ≤ 0.05.
